# The association between children’s exposure to pesticides and asthma, wheezing, and lower respiratory tract infections. A systematic review and meta-analysis

**DOI:** 10.3389/fpubh.2024.1402908

**Published:** 2024-05-24

**Authors:** Awoke Keleb, Chala Daba, Lakew Asmare, Fekade Demeke Bayou, Mastewal Arefaynie, Anissa Mohammed, Abiyu Abadi Tareke, Natnael Kebede, Yawkal Tsega, Abel Endawkie, Shimels Derso Kebede, Kaleab Mesfin Abera, Eyob Tilahun Abeje, Ermias Bekele Enyew

**Affiliations:** ^1^Department of Environmental Health, College of Medicine and Health Sciences, Wollo University, Dessie, Ethiopia; ^2^Department of Epidemiology and Biostatistics School of Public Health, College of Medicine and Health Science Wollo University, Dessie, Ethiopia; ^3^Department of Reproductive and Family Health, School of Public Health, College of Medicine and Health Sciences, Wollo University, Dessie, Ethiopia; ^4^Amref Health in Africa, COVID-19 Vaccine/EPI Technical Assistant at West Gondar Zonal Health Department, Gondar, Ethiopia; ^5^Department of Health Promotion, School of Public Health, College of Medicine and Health Sciences, Wollo University, Dessie, Ethiopia; ^6^Department of Health System and Management, School of Public Health, College of Medicine and Health Sciences, Wollo University, Dessie, Ethiopia; ^7^Department of Health Informatics, School of Public Health, College of Medicine and Health Sciences, Wollo University, Dessie, Ethiopia

**Keywords:** pesticide exposure, chronic respiratory diseases, asthma, respiratory tract infection, children, systematic review, meta-analysis

## Abstract

**Background:**

Exposure to pesticides is a global public health problem, especially for children. Its association with chronic respiratory disease among children has attracted considerable attention, but the existing evidence remains inconclusive and cannot be certain. Therefore, this systematic review and meta-analysis aim to determine the global pooled effect size of association with pesticide exposure and asthma, wheezing, and respiratory tract infections among children.

**Methods:**

A comprehensive search was conducted for relevant literature from electronic databases, including PubMed, Google Scholar, Hinari, Semantic Scholar, and Science Direct. Studies that provided effect size on the association between pesticide exposure and childhood asthma, wheezing, and respiratory tract infections in children were included. The articles were screened, data was extracted, and the quality of each study was assessed with four independent reviewers. Random effects models for significant heterogeneity and fixed effect models for homogeneous studies were conducted to estimate pooled effect sizes with 95% confidence intervals using Comprehensive Meta-Analysis version 3.3.070 and MetaXL version 2. Funnel plot and Higgins *I*^2^ statistics were used to determine the heterogeneity of the included studies. Subgroup analyses were computed based on the types of pesticide exposure, study design, sample size category, and outcome assessment technique.

**Result:**

A total of 38 articles with 118,303 children less than 18 years of age were included in this meta-analysis. Pesticide exposure among children increased the risk of asthma by 24%; (OR = 1.24, 95% CI: 1.14–1.35) with extreme heterogeneity (*I*^2^ = 81%, *p* < 0.001). Exposure to pesticides increased the odds of developing wheezing among children by 34% (OR = 1.34, 95% CI: 1.14–1.57), with high heterogeneity (*I*^2^ = 79%, *p* < 0.001) and also increased the risk of developing lower respiratory tract infection by 79% (OR = 1.79, 95% CI: 1.45–2.21) with nonsignificant low heterogeneity (*I*^2^ = 30%, *p*-value = 0.18).

**Conclusion:**

This meta-analysis provided valuable evidence supporting the association between childhood asthma, wheezing, and lower respiratory tract infection with pesticide exposure. The findings would contribute to a better understanding of the estimate of the effect of pesticide exposure on respiratory health in children and inform evidence-based preventive strategies and public health interventions.

## Introduction

In the 21st century, pesticide exposure continues to be a serious global public health concern, especially for children. Approximately 300,000 deaths per year are attributable to pesticide exposure, which affects about 3 million people globally (1). Data from poison control centers showed that 3.4% of pediatric deaths and 3.6% of adult deaths are attributable to pesticide poisoning, with 3.3% of unintentional poisoning deaths coming from all sources (2). It was indicated that children aged less than 19 years of age accounted for about 59% of all single-substance pesticide exposures, and 94% of all pesticide ingestions were done inadvertently. The associated costs of treating chronic illness are very substantial, especially in developing nations (1, 3).

There is a risk of accidental and occupational exposure to pesticide residues of many kinds, including pyrethroids, fungicides, organochlorine (OC), organophosphate (OP), (4), indoor metabolites, and other chemicals (5). Different studies indicated that mostly organophosphate pesticides can cause neurotoxicity, immune toxicity, genotoxicity, nephrotoxicity, hepatotoxicity, cardiotoxicity, and reproductive toxicity (6–8), while organochlorine and pyrethroid pesticides can cause both acute and chronic respiratory disease and allergic reactions (9–11).

Adult epidemiological research indicates that occupational and environmental exposure to pesticides is linked to a high incidence of respiratory illnesses and their symptoms (12), including asthma, wheezing, respiratory tract infections (13), and changes in lung function (10, 14, 15). According to the pooled prevalence from a meta-analysis of 56 publications, the ratio of forced expiratory volume in 1 s to forced vital capacity decreased as a result of exposure to organophosphate pesticides (16–19). This is also supported by three literature reviews that were recently published (9, 20, 21). However, little is known about the pooled effect size of exposure to pesticides and childhood chronic respiratory diseases, including asthma, wheezing, and other respiratory tract infections.

Exposure to pesticides has been associated with an increased risk of chronic respiratory diseases and symptoms in children, and they are particularly vulnerable to asthma, wheezing, and lower respiratory tract infections (20) due to their developing bodies, immune systems, and behaviors that may increase their exposure (22–25). Children are mostly exposed to pesticides through inhalation (compared to adults, children breathe more about their body weight) (10), consumption of food and drink that has a high pesticide residual content (17, 23), by skin contact or exposure while using pesticides at home to control pests (17), mothers’ exposure to pesticides during pregnancy, and their hand-to-mouth habit (25, 26).

Studies have examined the association between pesticide exposure and chronic respiratory diseases during childhood, and the previous studies conducted have not reached a consensus. This systematic review and meta-analysis aim to consolidate and determine pooled evidence on the association between chronic respiratory diseases and symptoms including asthma, wheezing, respiratory tract infection, and pesticide exposure. Understanding and pooled evidence for this relationship is crucial in advocating for environmental and occupational health regulations and promoting preventive measures to minimize the impact on public health.

## Methods

### Reporting system and registration

We used primary studies that reported the association between single or multiple pesticide exposure and asthma and/or LRTI and/ or wheezing and/or among children from prenatal to 18 years of age worldwide. Fundamental principles of the Centre for Reviews and Dissemination’s (CRD) guidance for undertaking reviews in healthcare and Preferred Reporting Items for Systematic Review and Meta-Analysis guideline (PRISMA) were employed to conduct this review. It was registered at the Protocols at the International Prospective Register of Systematic Reviews (PROSPERO: CRD42020176826) available.^1^

### Data sources, study period, searching strategies, and study selection

A comprehensive literature search was conducted in electronic databases, including PubMed, Google Scholar, Hinari, Semantic Scholar, and Science Direct. The search included studies published from the inception of the databases from 1991 up to December 2, 2023, and the studies included in the previous systematic review were reevaluated and incorporated in this meta-analysis.

An effort was made to get in touch with experts in the field to obtain further details about both published and unpublished research. In addition, relevant references in selected studies were examined thoroughly to find related studies that were not found in our search.

The MeSH and search filters were included in the search strategies (Pesticides OR Insecticides OR Organophosphate OR Carbamates) AND (Respiratory function OR Pulmonary function OR Respiratory symptoms OR Respiratory disease OR Respiratory Disorder OR Asthma OR Wheeze OR Bronchitis OR Dyspnea OR Cough OR Phlegm) AND Children (27). In addition to the above keywords, synonyms, abbreviated symbols, and other free keywords were used. Only full-text articles in the English language were considered for review, the reference lists were also manually checked, and similar articles feature of a database was used. The search was performed up to December 2, 2023, by four authors independently (AK, CD, YT, and ET).

Every included and excluded studies were screened using EndNote 20 and the Rayyan automation tool. Screening by title and abstract was conducted independently, followed by screening by the full texts of the included studies by four authors. Disagreement was solved by consensus, and the selection process was recorded in sufficient detail to complete a PRISMA 2020 flow diagram.

### Inclusion and exclusion criteria

In this review, cohort, case–control, or cross-sectional studies conducted (without restrictions to study period and sample size, study setting, and published and unpublished) on the association between pesticide exposure and chronic respiratory diseases, including asthma, lower respiratory tract infections (LRTI), and wheezing, among children less than 18 years of age. Observational studies conducted among children exposed to pesticides, insecticides, organophosphate, or carbamates and their derivatives were eligible for this review and compared with children who were not exposed with more or less exposed to pesticides, insecticides, organophosphate, carbamates, or their derivatives.

Children exposed to pesticides from agricultural sources, including parental and antenatal exposure, through air, via contaminated food, were also included, but studies carried out on groups other than children were excluded. Included outcomes were effect size reported on the association between asthma, wheezing, lower respiratory tract infection, and pesticide exposure among children less than 18 years of age. However, qualitative studies, irretrievable studies, editorial letters, studies with poor methodological quality, and studies that did not report the outcome of interest were excluded from the meta-analysis.

### Outcome assessment

The primary outcome of the study was to estimate the pooled effect size of the association between pesticide exposure and asthma, and LRTI and wheezing in the form of odds ratio.

### Data extraction quality assessment

After all, articles were exported into the EndNote 20 version and the Rayyan automation tool to remove the duplicated articles. The remaining data were extracted using a standardized form (initially piloted on two included studies) with characteristics of studies, outcomes, and risk of bias on Microsoft Excel 2016. Cohort, case–control, and cross-sectional studies using the author involved in the study, year, country, study design, sample size, type of pesticide exposed, exposure metrics, exposure assessment method, timing of outcome measurement, outcome assessment, and children with chronic respiratory disease (asthma and lower respiratory tract infections)/chronic respiratory symptoms (wheezing) associated with pesticide exposure were performed by five authors (AK, CD, YT, ET, and EB). After five reviewers (LA, AE, FD, MA, and AM) screened the relevant articles for eligibility, the quality of each article was evaluated using the Joana Brigg Institute (JBI) critical appraisal checklist (28). The four writers (AK, CD, AAT, and NK) evaluated the risk of bias for each study separately, and their scores were expressed on a 100% scale. A quality score of greater than 50% was used to include articles for further qualitative and quantitative analysis (28, 29). In the case of any discrepancies encountered during the quality assessment, the mean score was computed from the evaluations of all reviewers to address and resolve any differences.

### Data analysis and synthesis

All types of analysis were computed using Comprehensive Meta-Analysis (CMA) version 3.3.070 and MetaXL version 2. The pooled effect sizes of the association between asthma, wheezing, and pesticide exposure were calculated using the random effects models.

The *I*^2^ statistic was used to measure heterogeneity among the included studies and *I*^2^ values less than 50% were considered homogeneous, and *I*^2^ values greater than or equal to 50% were considered as of high heterogeneity. Begg’s funnel plots and Eggers test were employed to assess publication bias/small studies effect. A 95% Confidence Interval (CI) and *p*-value of less than 0.05 were considered significant for the association, absence of publication bias, and heterogeneity.

Subgroup analyses were conducted on different factors to identify sources of heterogeneity, including sample size (large vs. small), types of study design (case–control, cohort, cross-sectional), types of pesticide exposure (multiple vs. single), and types of outcome measurement (biomarker, doctor diagnosed, and self-reported) for asthma. Furthermore, to resolve heterogeneity further, sensitivity analysis was performed by removing one study in each scenario.

## Results

### Study selection and characteristics of the included studies

**Figure 1:**
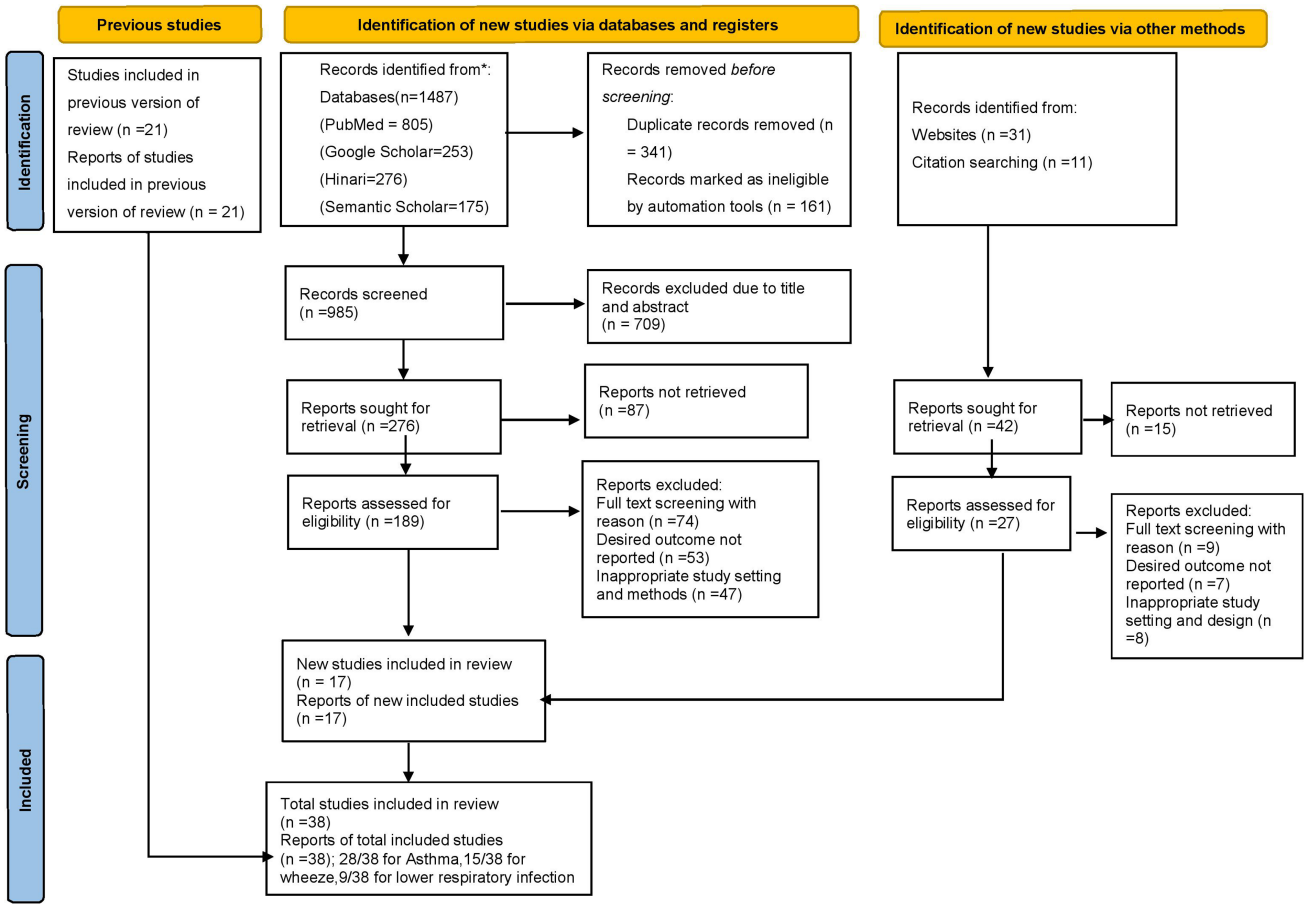
PRISMA flow diagram of the included studies for the systematic review and meta-analysis of respiratory diseases, 2023.

Based on the search study stated above, 1,487 studies from databases, 31 from websites, and 11 from citations were identified. A total of 502 studies from the database were discarded due to duplication. About 341 discarded studies were excluded via EndNote 20, and the remaining 161 studies were excluded using the Rayyan automation tool. Title and abstract parts of the remaining 985 studies were reviewed, of which 709 studies were excluded due to irrelevance. Out of the 276 studies that were sought to be retrieved, 87 could not be retrieved, and 189 were eligible for full-text screening. Finally, 15 studies from the new database, 2 studies from the website and citation, and 21 articles screened and reevaluated from previous reviews were eligible and included in the study. PRISMA flow chart related to the search process is shown in Figure 1.

### Characteristics of the included studies (qualitative review)

A total of 38 articles (23, 27, 30–66) were included to determine the association between exposure to pesticides, chronic respiratory diseases, and symptoms including asthma, wheezing, and respiratory tract infections. In this meta-analysis, a total 118,303 of children as study subjects were included. In this meta-analysis, 13 studies were carried out in United states of America (27, 31, 33, 35–38, 49–51, 53, 56, 66), four from Spain (34, 59–61), two from China (46, 63), two in Canada (43), two in South Africa (30, 39), three in Lebanon (44), two from Costa Rica (40, 47), one each from six countries (Italy, Germany, Romania, Netherland, one combined study from Greenland and Ukraine, and one combined study from North Europe and Australia) (32, 41, 42, 45, 48, 57), and one from England (62). Characteristics of all the included studies are summarized in Table 1.

**Table 1:** Characteristics of all the included studies based on the pesticide exposure and respiratory health outcomes, 2023.

Author	Year	Country	Study design	Study sample	Pesticide addressed	Exposure metrics	Exposure assessment method	Timing of outcome measurement	Outcomes assessment method	Health effects and its association
Benka-Coker. et al.	2019	USA	Cohort	16	OP	agriculture	urine samples for DAP (summative measures)	Not clear	Biomarkers (Urinary LTE)	Asthma 8.7(95%CI:3.512,14.600)
Bukalasa et al.	2016	Netherlands	Cohort	1,470	Multiple exposure	agriculture	Questionnaire interview	0–14 years old	Self-reported	Asthma OR = 0.860, 95%CI:0.520, 1.400 with 1KM distance Respiratory symptoms OR = 0.860, 95%CI:0.520, 1.400 with 1KM distance
Elsiwi et al.	2023	South Africa	Cohort	620	Pyrethroid	environmental	maternal urine 3PBA	up to 5 years	Doctor diagnosed	Asthma, (OR = 2.400, 95%CI:1.000, 5.800) Wheeze, OR = 1.80 95%CI: 1.00, 3.30
Famid et al.	2020	South Africa	Cohort	652	Organochlorine	Prenatal	Cord blood DDT	Up to 3.5 years	Doctor diagnosed	Wheeze, (OR = 1.500, 95%CI:1.000, 2.300)
Gascon et al.	2007	Spain	cohort	405	DDE/OC	environmental	cord blood DDE+ immune biomarker	birth to 14 years old	doctor diagnosed	Asthma 10 years, RR = 1.03 (95%CI: 0.71, 1.50) 14 years, RR = 0.89 (95%CI: 0.61, 1.31) Wheeze 10 years, RR = 1.22 (95%CI: 0.91, 1.63) 14 years, RR = 0.92 (95%CI: 0.64, 1.31) LRTI 10 years, RR = 1.27 (95%CI: 0.86, 1.86)
Gharibi et al. a	2019a	USA	cross sectional	4,262	methyl bromide	environmental	questionnaire interview	2–18 years old	doctor diagnosed	Asthma OR = 1.02, 95% CI: 0.99, 1.05, for 2–5 years OR = 1.07, 95% CI: 1.02, 1.12 for 6–18 years
Gharibi et al. b	2019b	USA	cross sectional	1,331	1,3-dichloropropene	environmental	questionnaire interview	2–18 years old	doctor diagnosed	Asthma (OR = 1.06, 95%CI: 1.02, 1.11 OR = 1.14, 95%CI: 1.07, 1.19)
Gilden et al.	2020	USA	Cohort	390	OP and pyrethroid	Prenatal	urinary DAP and 3PBA	Up to 8 years old	interview and spirometry	Wheeze OR = 2.32, 95%CI: 1.28, 4.17
Gunjer et al.	2018	USA	Cohort	294	multiple pesticides	Agriculture	questionnaire interview	prenatal to 7 years	Interview and Spirometry	Asthma OR = 3.60, 95%CI: 1.04, 12.10
Halit et al.	2017	Lebanon	case control	1,503	multiple pesticides	environmental	questionnaire interview	3–16 years old	self-reported	Asthma OR = 2.709, 95%CI: 1.219, 6.020 LRTI OR = 2.71 (95%CI: 1.22, 6.02)
Huq et al.	2020	South Africa	Cohort	658	multiple	Prenatal	Questionnaire	3.5 years	Doctor diagnosis	Wheeze OR = 1.500 (95%CI: 1.000, 2.300)
Islam et al.	2016	Costa Rica	cross sectional	303	Pyrethroid	Agriculture	questionnaire interview	up to 5 year	Doctor diagnosed	Asthma OR = 2.090, 95%CI: 0.980, 4.360 Wheeze OR = 2.37(95%CI: 1.28, 4.34) LRTI OR = 2.78 (95%CI: 0.41, 8.04)
Kuramaus et al.	2001	Germany	cross sectional	343	OC/DDE	Agriculture	cord blood DDE	3–7 years old	Self-reported	Asthma OR = 3.710, 95%CI: 1.100, 12.560,
Kuramaus et al.	2003	Germany	cross sectional	338	OC/DDE	Agriculture	cord blood DDE	7–10 years old	Self-reported	Asthma OR = 3.040, 95%CI: 0.530, 22.300,
Lu et al.	2018	Romania	cross sectional	280	multiple pesticides	environmental	questionnaire interview	6–11 years old	self-reported	Multi-pollutant controlled to CO2 vs. asthma OR = 4.17 (5%CI:1.430, 13.260)
Malaeb et al.	2020	Lebanon	cross sectional	1,203	multiple pesticides	environmental	questionnaire interview	4–7 years	self-reported	Wheeze OR = 2.06(95%CI: 1.08, 3.91)
Maritano et al.	2016	Italy	case control	5,346	multiple pesticides	environmental	questionnaire interview	6–18 months	self-reported	Wheeze OR = 1.72(95%CI: 1.11, 2.65)
Masley et al.	2000	Canada	cross sectional	393	multiple pesticides	Agriculture	questionnaire interview	Up to 17 years old	self-reported	Asthma OR = 2.200, 95%CI: 0.800, 5.700 Wheeze OR = 2.20(95%CI: 0.80,6.02) Bronchitis/RTI OR = 2.80 (95%CI: 1.60, 4.80)
Meng et al.	2016	China	case control	620 case/218 control	OC	environmental	questionnaire interview	3–6 years	doctor diagnosed	Severe Asthma OR = 1.000, 95%CI:0.99, 1.002,
Mora et al.	2020	Costa Rica	Cohort	355	Fungicides	Prenatal	Seven Urinary metabolites	1st trimester to 19 months of postpartum	Biomarker	Wheezing OR = 0.69 (95%CI: 0.37, 1.28) LRTI OR = 1.500, 95%CI:0.70, 3.19,
Pape et al.	2020	North Europe & Australia	Cohort	2,766	Multiple exposure b	prenatal and environmental	Questionnaire interview	0–15 years old	Self-reported	Asthma OR = 1.180, 95%CI:0.870, 1.610
Perla et al.	2016	USA	cross sectional	10,077	OP and DDT	environmental	blood and urine DAP test	Up to 16 years	Biomarker	Ever Asthma >75^th^ percentile RR = 1.160, 95%CI: 0.620, 2.170,
Raanan et al.	2015	USA	Cohort	342	OP/DAPs	Agriculture	Maternal interview& urinary DAPs	prenatal up to 7 years	Self-reported respiratory symptoms	Respiratory symptoms OR = 2.530, 95%CI:1.320, 4.86, from children
Raanan et al.	2017	USA	Cohort	347	Elemental sulfur	agriculture	Pesticide use report	Short term exposure	Self-report	Respiratory symptoms/wheeze (OR = 2:09; 95% CI: 1.27; 3.46, p = 0.004) with 1KM distance
Raherison et al.	2019	France	cross sectional	281	multiple pesticides	environmental	urine ETU and air monitoring	not clear	Biomarker and clinical	Pesticide in air vs. Asthma OR: 3.930, 95% 0.400–38.440 Urinary ETU vs. Asthma OR = 2.010, 95%CI: 0.540, 7.520
Reardon et al.	2009	USA	Cohort	652	OP + Pyrethroids	Agriculture	questionnaire interview	prenatal to 12 months	Biomarkers	Wheeze (OR = 0.83,95%CI: 0.72–0.95)
Salam et al.	2004	USA	case control	4,000	multiple pesticides	environmental	questionnaire interview	prenatal to 12 months	doctor diagnosed	Asthma (OR = 1.61; 95% CI, 0.930–2.790)
Salameh et al.	2003	Lebanon	cross sectional	3,291	multiple pesticides	environmental	questionnaire & residential exposure score	3 years −16 years	self-reported	Asthma (OR = 1.73; 95%CI: 1.02–2.97), Respiratory disease/RTI (OR = 1.71; 95%CI: 1.20–2.43), Ever wheezing (OR = 1.99; 95%CI: 1.43–2.78)
Smit et al.	2016	Greenland & Ukrain	Cohort	1,024	OC/PCB 153	environmental	blood sample PCB	5–9 years	doctor diagnosed	Asthma, (OR = 0.960, 95%CI:0.770, 1.200) Wheeze,(OR = 0.840, 95%CI:0.700, 1.010)
Sunyer et al.	2005	Spain	Cohort	468	OC/DDE	Agriculture	cord blood DDE	at age 4 year	Doctor diagnosed	Wheeze OR = 1.32, 95%CI:1.130, 1.540
Sunyer et al.	2006	Spain	Cohort	402	OC/DDE	Agriculture	cord blood DDE	at age 6.5 year	Doctor diagnosed	asthma (OR = 1.180, 95%CI:1.01, 1.39) Wheeze,(OR = 1.130, 95%CI:0.980, 1.300)
Sunyer et al.	2010	Spain	Cohort	584	OC/DDE	Prenatal	Maternal serum	6–14 months	Doctor diagnosis	RTI/LRTI (OR = 2.40, 95%CI:1.19, 4.83)
Tagiyeva et al. 2010	2010	England	Cohort	13,971	Biocides and fungicides	Maternal postnatal exposure	Maternal interview	0–8.5 years	Doctor diagnosis	Asthma (OR = 1.470, 95%CI: 1.100, 1.880)
Wang et al.	2021	China	cross sectional	627	Insecticide	Residential	survey using questionnaire	school children	interview and spirometry	Asthma OR = 2.128 (95%CI: 0.796, 5.689) Bronchitis/RTI OR = 2.05 (95%CI: 1.38, 3.05)
Werthmann et al.	2023	USA	Cohort	162	multiple pesticides	environmental	Urinary pesticide metabolite (3,PBA)	7–12 years	doctor diagnosed	Asthma (OR = 1.070, 95%CI: 0.880, 1.290)
Weselak et al.	2007	Canada	Cohort	3,405	multiple pesticides	Agriculture	questionnaire interview	Prenatal	self-reported	Any pesticide vs. Asthma (OR = 1.000, 95%CI: 0.710, 1.400) Bronchitis/RTI OR = 1.21 (95%CI: 0.77, 1.90)
Xiao et al.	2021	USA	cross sectional	41,423	multiple pesticides	household	survey using questionnaire	up to 17 year	self- reported	Asthma OR = 1.070 (95%CI: 0.800, 1.420)
Xu et al.	2014	USA	cross sectional	14,065	multiple pesticides	Residential	questionnaire interview	1–17 years old	self-reported	Wheeze OR = 1.390 (95%CI: 1.080, 1.780)

### The association of pesticide exposure and childhood asthma

**Figure 2:**
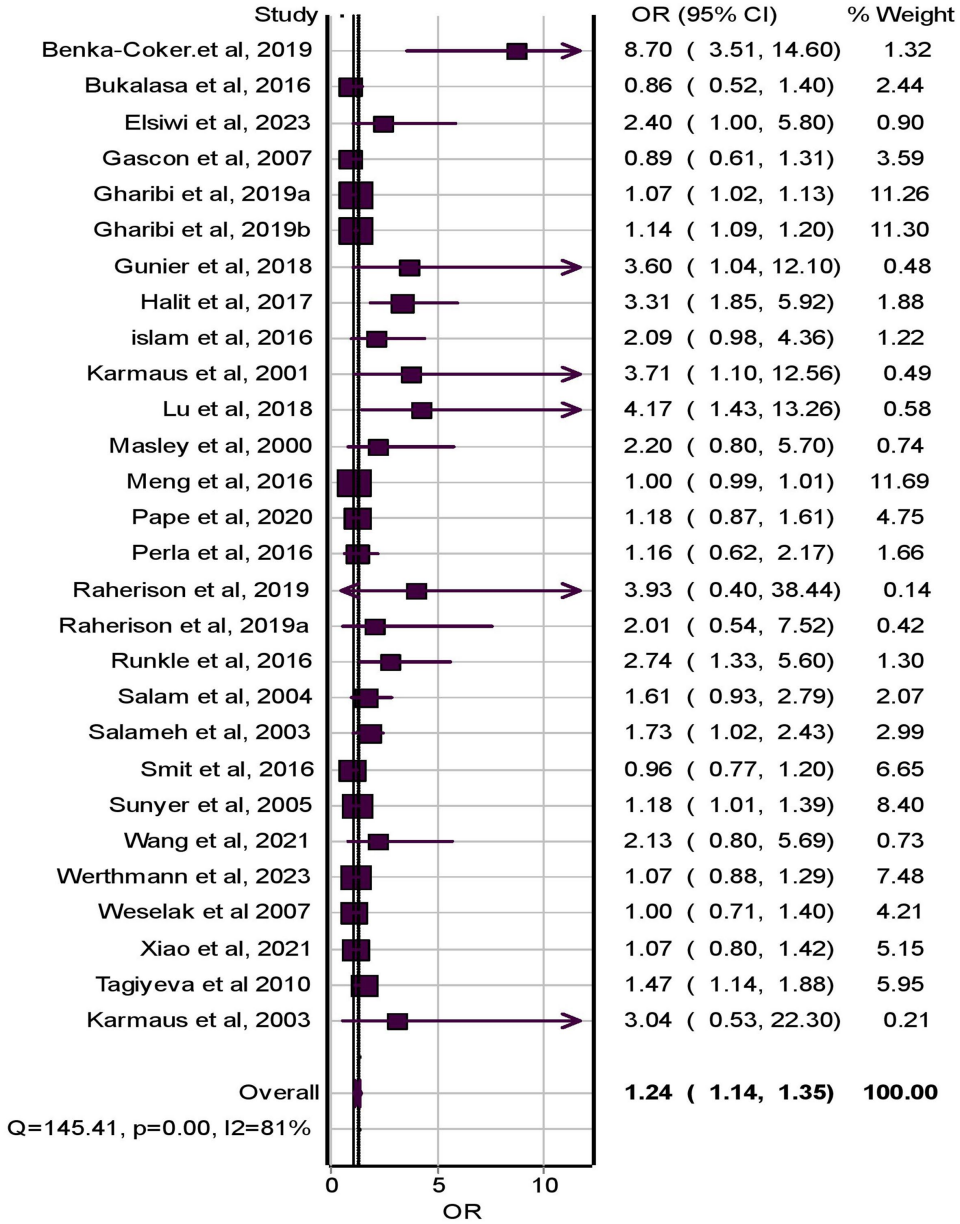
Forest plot of odds ratios for the association of pesticide exposure and childhood asthma, 2023.

A total of 28 studies (27, 30, 32–36, 38, 41–43, 46, 48, 49, 52, 54–58, 60, 63, 64) were included in the random effect model meta-analysis to examine the association between childhood asthma and pesticide exposure. Pesticide exposure had a statistically significant association between childhood asthma and pesticide exposure with pooled effect size, (OR = 1.24, 95% CI = 1.14–1.35, *p*-value <0.001) with significant extreme heterogeneity (I^2^ = 81%, *p* < 0.001) among included studies (Figure 2).

### The association of pesticide exposure and childhood wheeze

**Figure 3:**
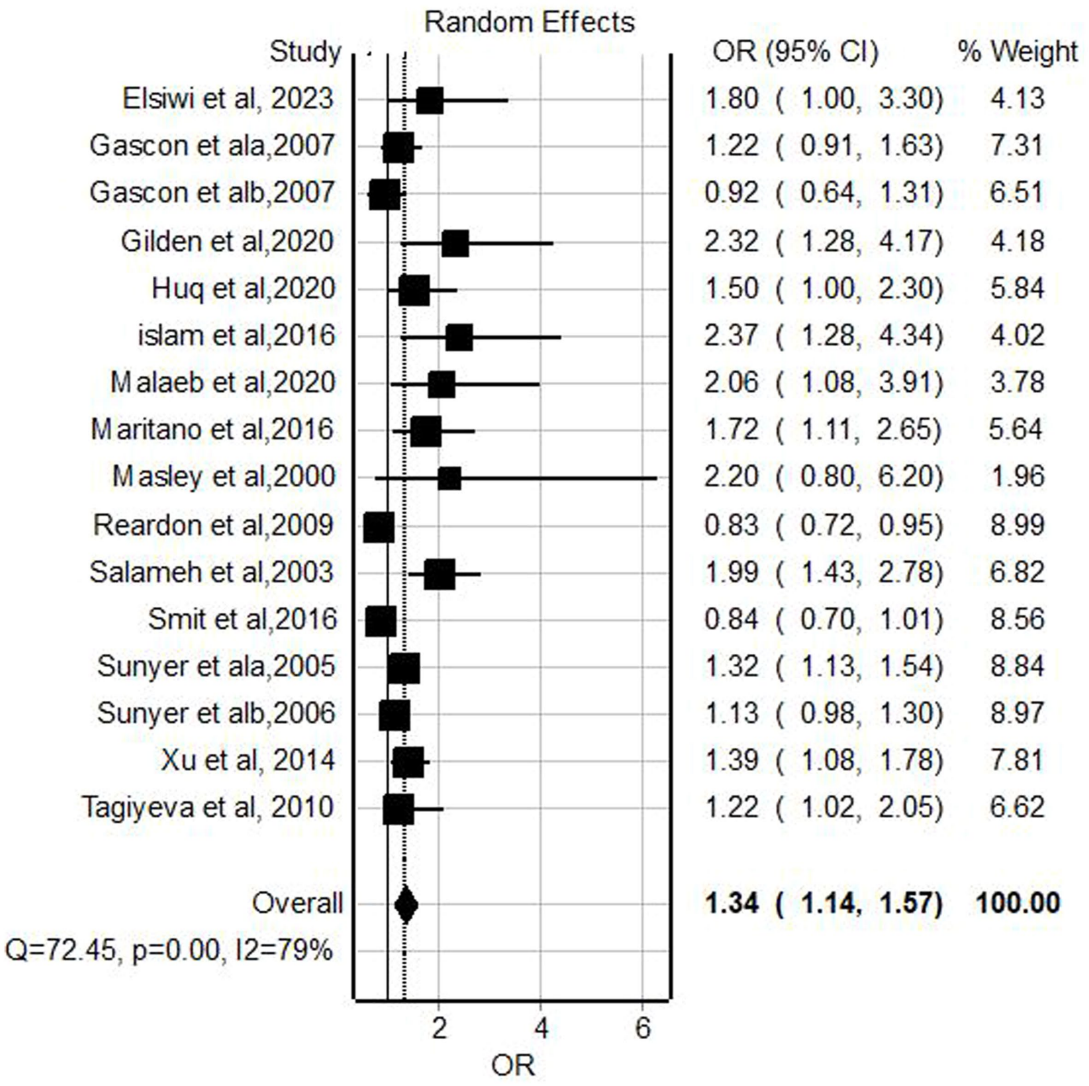
Forest plot of odds ratios for the association of pesticide exposure and childhood wheeze, 2023.

Fifteen studies (30, 34, 37, 39, 40, 43–45, 53, 55, 57, 60, 61, 66) were included in the meta-analysis for the association between pesticide exposure and wheezing among children. The result from the random effect model indicated that pesticide exposure had a statistically significant association with the occurrence of childhood wheezing (OR = 1.34, 95% CI = 1.14–1.57, *p*-value <0.001) and significant extreme heterogeneity (I^2^ = 79%, *p* < 0.001) within included studies (Figure 3).

### The association of pesticide exposure and lower respiratory tract infections

**Figure 4:**
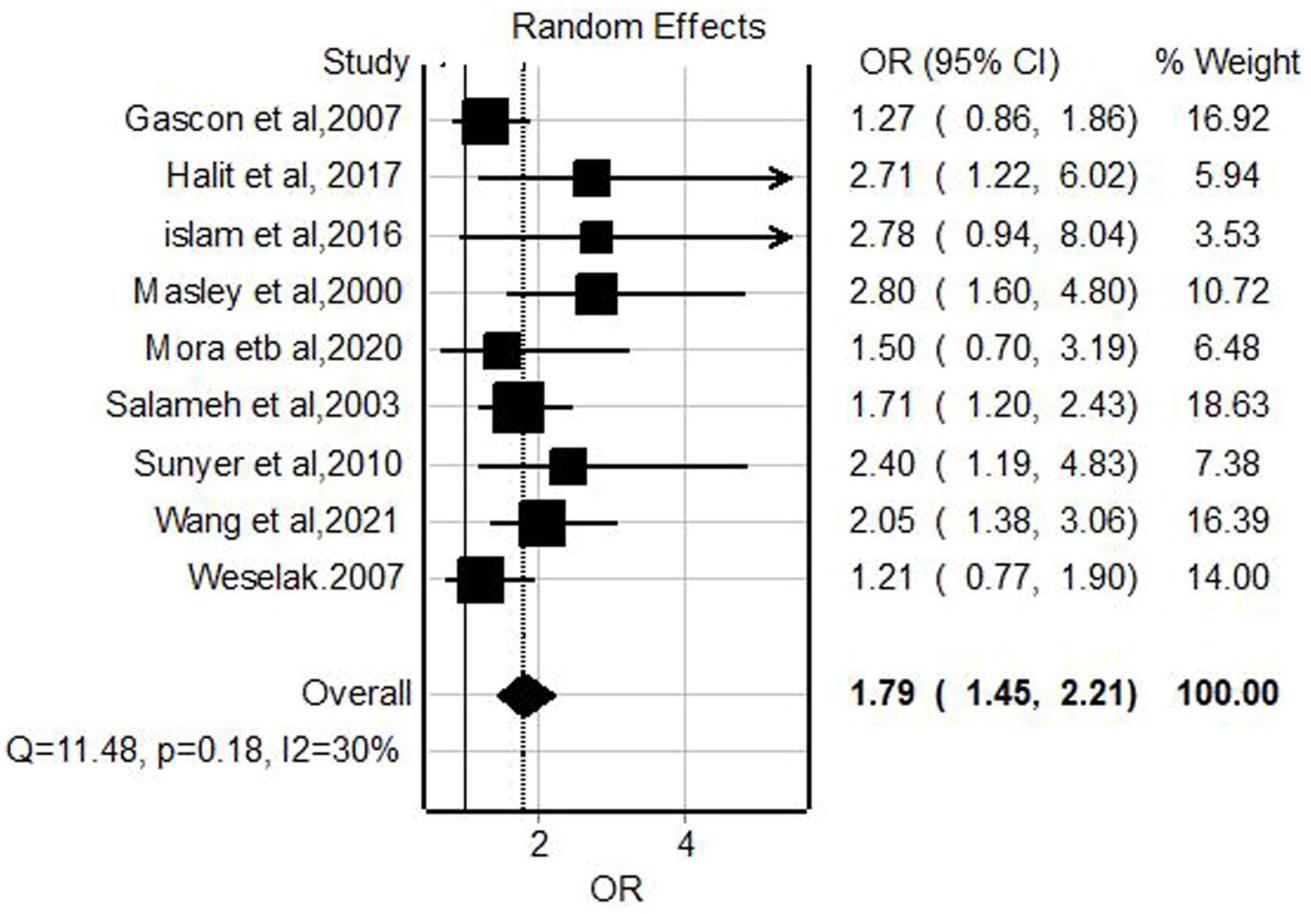
Forest plot of odds ratios for the association pesticide exposure and lower respiratory infections among children, 2023.

Meta-analysis of nine relevant studies (34, 40, 43, 47, 55, 58, 59, 63, 64) using fixed effect model showed a significant association between lower respiratory tract infections and pesticide exposure with a pooled odd ratio of 1.79 (95% CI = 1.45–2.21, *p*-value <0.001) with nonsignificant low heterogeneity (*I*^2^ = 30%, *p*-value = 0.18; Figure 4).

### Publication bias

**Figure 5:**
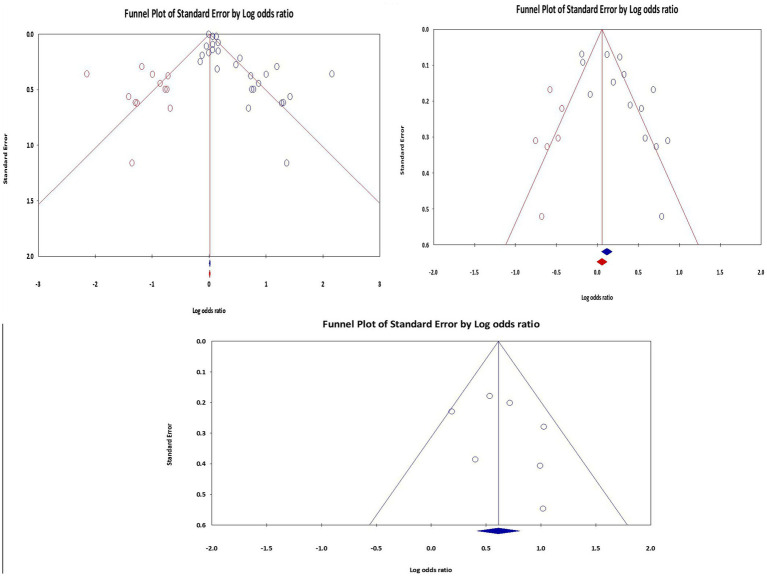
Funnel plot of association of pesticide exposure and asthma, wheeze and LRTI, 2023.

Publication bias occurs when research with significant results is more likely to be published than those with nonsignificant results. This bias may cause effect sizes to be overestimated and an inflated perception of the strength of the association. To mitigate the possibility of selective publication, a thorough literature search utilizing multiple databases was carried out to reduce publication bias. Egger’s test and Begg’s funnel plots were used to evaluate any potential publication bias quantitatively. For wheezing and asthma, publication bias was found (*p* value <0.05). To estimate the effect size by imputing or “filling in” potentially missing data, trim and fill analysis was carried out on the left using a fixed model (adjusted values from 12 trimmed studies with a point estimate of 1.01, with 95% CI = 1.00–1.02 for asthma and 6 trimmed studies with a point estimate of 1.16. with 95% CI: 1.08–1.24 for wheeze). However, publication bias was not detected in the case of lower respiratory infection (*p*-value = 0.403) during meta-analysis (Figure 5).

### Subgroup analyses for asthma

Statistically significant association (OR = 1.32, 95% CI, 1.15–1.51, *p* < 0.001) with extreme heterogeneity (*I*^2^ = 85.6%, *p* < 0.001) was detected among studies with small sample sizes (<1,000). The effect sizes between pesticide exposure and childhood asthma vary moderately for both cohorts, (OR = 1.19, 95% CI: 1.02–1.39, *p* = 0.025, with I^2^ = 75.9%, *p* < 0.001) and cross-sectional studies (OR = 1.35, 95% CI: 1.14–1.59, *p* = 0.001, with I^2^ = 65.2%, *p* < 0.001) in contrast to the nonsignificant association for case–control studies (Supplementary Table S1).

Studies investigating multiple pesticide exposures reported a significant association of 1.27 (95% CI: 1.10–1.94, *p* = 0.002) with moderate significant heterogeneity (*I*^2^ = 58.8%, *p* < 0.000), whereas studies focused on single pesticide exposures found a smaller but still significant effect size of 1.16 (95% CI: 1.05–1.29, *p* = 0.004) with higher significant heterogeneity (*I*^2^ = 85.6%, *p* < 0.001) among the included studies (Table 2).

**Table 2:** Subgroup analysis of the pooled effect size and heterogeneity of asthma among children exposed to pesticide globally, 2023.

Variable	Category	No of studies	Effect size and significance		Heterogeneity	
			OR (95%CI)	*p*-value	I^2^	*p*-value
Sample size	Large(≥1,000)	11	1.14(0.99–1.32)	0.077	36.7%	0.115
	Small(<1,000)	17	1.32(1.15–1.51)	<0.000	85.6%	<0.000
Study design	Case control	3	1.21(0.92–1.60)	0.174	77.4%	0.012
	Cohort	13	1.19(1.02–1.39)	0.025	75.9%	<0.000
	Cross sectional	12	1.35(1.14–1.59)	0.001	65.2%	0.001
Types of exposure	Multiple pesticide	13	1.27(1.09–1.47)	0.002	58.8%	0.005
	Single pesticide	15	1.16 (1.05–1.29)	0.004	85.6%	<0.000
Outcome measurement	Biomarkers	7	1.19(0.96–1.48)	0.110	54.5%	0.040
	Doctor diagnosed	13	1.18(1.05–1.34)	0.004	87.9%	<0.000
	Self-reported	7	1.23(1.05–1.45)	0.011	39.7%	0.077

### Sensitivity analysis

A sensitivity analysis was carried out to assess the robustness of the meta-analysis findings. The total effect size estimates were consistent and statistically significant, even after individual trials were systematically removed to evaluate their impact. Removing studies with a high potential for bias had no discernible impact on the findings. The pooled effect size estimate held true after accounting for publication bias, which was evaluated. However, it is imperative to consider data limitations and potential sources of heterogeneity when interpreting the results.

## Discussion

Pesticides, such as fungicides, insecticides, and herbicides, have been widely used since the 1950s to increase crop yields (67). Exposure to these substances raised knowledge of the risks associated with respiratory diseases and/or symptoms (14, 16, 19, 68). This review demonstrates that numerous epidemiological studies showed an association between children’s exposure to pesticides (from household, prenatal, postnatal, caregiver agricultural activities, residential, and environmental sources) and an increased risk of developing respiratory tract infections, asthma, and wheezing.

Because of the nature of the study design or the use of retrospective questionnaires, the measurement of pesticide exposure is frequently restricted in particular studies. However, 16 studies assessed the levels of pesticide metabolites in blood and urine, which is a more trustworthy estimate than utilizing a questionnaire during an interview.

The objective of this systematic review and meta-analysis aimed to determine the pooled effect size between prenatal, occupational, and environmental pesticide exposure and chronic respiratory disease/symptoms including asthma, lower respiratory tract infection, and wheezing globally. A significant association was found between pesticide exposure and asthma among children in this review aligns with previous systematic reviews and meta-analyses that have reported positive associations between pesticide exposure and asthma (18–21, 69). These concordant results across different meta-analyses strengthen the evidence for the association and underscore its significance in the field (22, 23, 70), and two recent studies with new perspectives have investigated the association between exposure to pesticides in indoor dust and respiratory outcomes including asthma and wheezing (5, 71). However, Mthethwa et al. (72) found that inconsistent patterns of increased risk of asthma outcomes with increasing organophosphate concentrations among school children, and Gunier et al. (38) also reported a negative association between pesticide exposure and childhood asthma.

The subgroup analysis of childhood asthma within this study revealed significant variability among the included studies. The general possible reasons for this variation seen in the study might include differences in exposure assessment techniques (self-reporting, biomarkers, doctor diagnosed, or environmental monitoring), outcome ascertainment (diagnostic criteria and follow-up durations), populations’ characteristics (demographics, geographic locations, occupational exposures, and underlying health conditions), and publication bias, as well as variations in study designs, such as cohort, case–control, or cross-sectional studies.

Significant heterogeneity might impact the interpretation and generalizability of meta-analytic results; hence subgroup analysis was computed very carefully on types of study design, sample size, level of exposure, and method of measurement to produce subgroup effect sizes that can be interpreted in the context of the observed variability.

A significant level of heterogeneity among studies characterized by small sample sizes may be because small sample sizes lead to less precise estimates and greater susceptibility to chance variations or biases. Additionally, differences in exposure assessment, outcome measurement, children’s characteristics, and study design across these studies may contribute to the observed heterogeneity.

A significant heterogeneity across cohort and cross-sectional studies in this meta-analysis can be attributed to several factors, including temporal limitations in establishing causality, children’s characteristics such as age distribution, genetic predisposition, and environmental factors, potential bias, and unmeasured confounding variables.

Cross-sectional studies may encounter limitations in establishing temporal relationships due to the simultaneous assessment of exposure and outcome, making it challenging to determine the direction of causality accurately. Despite significant differences across the studies, 13 birth cohort studies with fewer recall bias and better control of confounders strongly suggest a causal association between pesticide exposure and asthma in children.

Both multiple- and single-pesticide exposure studies showed significant heterogeneity within each exposure category, highlighting differences between studies focusing on multiple versus single exposures. This variation might be attributed to differences in the types and combinations of pesticides across regions, individual susceptibility influenced by genetics and lifestyle, as well as variations in study design and methodology. These factors underscore the complexity of understanding the association between pesticide exposure and asthma among children.

The current meta-analysis also grouped studies according to how they measured the outcome (asthma) and found substantial variability between doctor-diagnosed asthma and biomarker measurements. This difference may stem from differences in diagnostic accuracy and variability in the interpretation of diagnostic criteria. Biomarkers provide objective measures of asthma, but there may still be variability in their use and interpretation across studies. On the other hand, doctor-diagnosed asthma relies on subjective assessment, which can vary greatly depending on individual clinician judgment, diagnostic criteria, and healthcare settings. These differences enforce to underscore the importance of standardized diagnostic approaches in research.

On the other hand, studies utilizing self-reported measures of asthma demonstrated nonsignificant heterogeneity. This may be because, in comparison to biomarker or doctor-diagnosed methods, self-reported measures of asthma typically capture a wider range of symptoms and experiences directly from individuals, which may result in higher sensitivity for detecting associations. Additionally, self-reported measures are typically more accessible and less resource-intensive, potentially leading to larger sample sizes and increased statistical power. Furthermore, a lower level of heterogeneity indicates more consistency in the methods used across studies employing self-reported measures, such as standardized questionnaires or protocols. However, it is important to acknowledge that reliance solely on self-reporting may introduce bias due to misclassification or recall errors, which could impact the observed effect sizes.

These subgroup findings also emphasize the need for further investigation and targeted research in specific children’s age categories and follow-up. Future studies on pesticide exposure and childhood asthma need to focus on specific age cohorts, use rigorous methodology, and account for different variances. However, the inclusion of a large number of studies appropriately accounts for the observed heterogeneity between studies, which increases the generalizability of the findings. This comprehensive approach also enhances our understanding of the association between pesticide exposure and childhood asthma and emphasizes targeted preventive measures and other public health interventions.

A significant association between wheeze and pesticide exposure of this finding aligns with the previous studies that have reported positive associations between pesticide exposure and wheezing among children (19, 20, 25, 37, 44, 73), particularly, in residential, agricultural, environmental, and prenatal exposure (23, 68, 73) and school (71). However, the included studies had statistically significant high heterogeneity, which was highly contrasted from study to study.

The variations in findings across different studies may be attributed to methodological differences, such as variations in study design, sample size, and age category of children or differences in exposure assessment methods including self-reporting or biomarker measurements. Furthermore, variations in study populations, including demographic characteristics and occupational backgrounds of mothers, caregivers, and fathers of children, may also influence the observed associations.

This meta-analysis also identified a significant positive association between pesticide exposure and an increased risk of lower respiratory tract infections with nonsignificant low heterogeneity. A significant association between pesticide exposure and lower respiratory tract infections from this finding is consistent with several previous studies that have reported positive associations between pesticide exposure and LRTI among children (19, 23, 40, 45, 47, 60). However, some studies have reported contrasted results (74) potentially due to methodological differences, variations in exposure assessment, and study populations. Finally, the included studies indicated non-significant heterogeneity that did not vary from study to study.

## Limitation and strength of the study

We identified several limitations on the reporting of windows of susceptibility, timing, length, and surrogates of exposure assessment. The majority of studies relied on questionnaires and self-reported exposure, which can be affected by recall bias and exposure misclassification. However, this meta-analysis followed the updated preferred reporting items for systematic review and meta-analysis. In this meta-analysis, all cases including asthma, wheezing, and lower respiratory tract infection as a result of exposure to pesticides were appropriately assessed.

## Conclusion

This meta-analysis showed that children who are exposed to pesticides are at an increased risk of developing chronic respiratory diseases and symptoms specifically asthma, RTI, and wheezing. This is particularly concerning as respiratory health problems could have long-term effects on a child’s health and well-being. Parents and other caregivers who care for children must understand the possible dangers of pesticide exposure and take precautions to reduce their exposure to these dangerous substances.

This can involve utilizing natural pest management techniques, selecting organic products, and pushing for stronger laws governing the use of pesticides in agricultural operations. Policymakers must take a leading role in safeguarding children from pesticide exposure by implementing regulations and policies that prioritize the health and safety of our most vulnerable populations to ensure that they have the opportunity to grow up in a healthy and safe environment.

Future research on pesticide exposure and asthma, wheezing, and LRTI among children should focus on longitudinal studies with an accurate assessment of pesticide exposure to capture cumulative long-term effects. Novel methods have been used to investigate the combined health effects of multiple pesticide exposures. Stratified analyses can also elucidate susceptibility factors, while mechanistic studies and metabolomics are essential for uncovering the biological pathways of pesticide toxicity.

Evaluating the effectiveness of preventive measures through intervention studies is better to inform strategies to mitigate respiratory health risks associated with pesticide exposure among children. Moreover, international research protocols tailored to local specificities should be developed and validated to compare studies conducted in different settings and enhance our understanding of the complexities of pesticide exposure and respiratory health outcomes among children.

## Data availability statement

The original contributions presented in the study are included in the article/supplementary material, further inquiries can be directed to the corresponding author.

## Author contributions

AK: Conceptualization, Data curation, Formal analysis, Methodology, Software, Supervision, Validation, Visualization, Writing – original draft, Writing – review & editing. CD: Conceptualization, Data curation, Formal analysis, Methodology, Software, Supervision, Validation, Writing – original draft, Writing – review & editing. LA: Data curation, Investigation, Methodology, Software, Supervision, Writing – original draft, Writing – review & editing. FB: Conceptualization, Data curation, Methodology, Software, Supervision, Validation, Writing – original draft, Writing – review & editing. MA: Conceptualization, Data curation, Formal Analysis, Methodology, Software, Supervision, Validation, Writing – original draft, Writing – review & editing. AM: Conceptualization, Data curation, Formal analysis, Methodology, Software, Supervision, Validation, Writing – original draft, Writing – review & editing. AT: Conceptualization, Data curation, Methodology, Software, Supervision, Visualization, Writing – original draft, Writing – review & editing. NK: Conceptualization, Data curation, Formal analysis, Methodology, Software, Validation, Visualization, Writing – original draft, Writing – review & editing. YT: Conceptualization, Data curation, Formal analysis, Investigation, Methodology, Software, Supervision, Validation, Visualization, Writing – original draft, Writing – review & editing. AE: Conceptualization, Data curation, Formal analysis, Methodology, Software, Supervision, Validation, Writing – original draft, Writing – review & editing. SK: Writing – original draft, Writing – review & editing. KA: Conceptualization, Data curation, Methodology, Resources, Software, Supervision, Validation, Writing – original draft, Writing – review & editing. EE: Conceptualization, Data curation, Methodology, Software, Supervision, Validation, Visualization, Writing – original draft, Writing – review & editing. EB: Conceptualization, Data curation, Formal analysis, Methodology, Software, Supervision, Validation, Writing – original draft, Writing – review & editing.

## Footnotes

^1^ https://www.crd.york.ac.uk/Prospero
