# Supplementary material for: The association between children’s exposure to pesticides and asthma, wheezing, and lower respiratory tract infections. A systematic review and meta-analysis
Source: Front Public Health. 2024 May 24;12:1402908. doi: 10.3389/fpubh.2024.1402908 (PMC11167956; doi:10.3389/fpubh.2024.1402908)
Supplement: Supplementary file 1 [file Table_1.docx]

**SM Table 1: quality assessment result of each study using JBI checklist**

**JBI for Cohort studies**

| Studies | Were the two groups similar & recruited from the same population? | Exposed & unexposed measured similarly | exposure measured in a valid & reliable way | confounding factors identified | strategies for confounding factors stated? | free of the outcome at the start of the study | outcomes measured in a valid and reliable way | follow up time reported & sufficient to be long enough | Complete  follow up | | strategies to address incomplete follow up utilized | appropriate statistical analysis used | Total score | Level of bias |
| --- | --- | --- | --- | --- | --- | --- | --- | --- | --- | --- | --- | --- | --- | --- |
| Benka-Coker.et al | Yes | Yes | Yes | Yes | Yes | Yes | Yes | Yes | Yes | No | | Yes | 10 | 90.9% |
| Bukalasa et al | Yes | Yes | Yes | Yes | Yes | Yes | Yes | Yes | Yes | No | | Yes | 10 | 90.9% |
| Elsiwi et al | Yes | Yes | Yes | No | No | Yes | Yes | Yes | Yes | No | | Yes | 8 | 73.7% |
| Famid et al | Yes | Yes | Yes | Yes | Yes | Yes | Yes | Yes | Yes | No | | Yes | 10 | 90.9% |
| Gascon et al | Yes | Yes | Yes | Yes | Yes | Yes | Yes | Yes | Yes | No | | Yes | 10 | 90.9% |
| Gilden et al | Yes | Yes | Yes | Yes | No | Yes | Yes | Yes | Yes | No | | Yes | 9 | 81.8% |
| Gunjer et al | Yes | Yes | Yes | Yes | No | Yes | Yes | Yes | Yes | No | | Yes | 9 | 81.8% |
| Huq et al | Yes | Yes | Yes | Yes | Yes | Yes | Yes | Yes | Ye | No | | Yes | 9 | 81.8% |
| Islam et al | Yes | Yes | Yes | Yes | No | Yes | Yes | Yes | Yes | Yes | | Yes | 10 | 90.9% |
| Maritano et al | Yes | Yes | Yes | Yes | No | Yes | Yes | Yes | Yes | No | | Yes | 9 | 81.8% |
| Mora et al | Yes | Yes | Yes | Yes | No | Yes | Yes | Yes | Yes | Yes | | Yes | 10 | 90.9% |
| Pape et al | Yes | Yes | Yes | Yes | Yes | Yes | Yes | Yes | Yes | No | | Yes | 10 | 90.9% |
| Raanan et al a | Yes | Yes | Yes | Yes | Yes | Yes | Yes | Yes | Ye | No | | Yes | 10 | 90.9% |
| Raanan et al b | Yes | Yes | Yes | Yes | Yes | Yes | Yes | Yes | Ye | No | | Yes | 10 | 90.9% |
| Reardon et al | Yes | Yes | Yes | Yes | Yes | No | Yes | Yes | Yes | Yes | | No | 9 | 81.8% |
| Smit et al | Yes | Yes | Yes | Yes | No | Yes | Yes | Yes | Ye | No | | Yes | 9 | 81.8% |
| Sunyer et al a | Yes | Yes | Yes | Yes | Yes | Yes | Yes | Yes | Ye | No | | Yes | 9 | 81.8% |
| Sunyer et al b | Yes | Yes | Yes | Yes | No | Yes | Yes | Yes | Ye | No | | Yes | 10 | 90.9% |
| Sunyer et al c | Yes | Yes | Yes | Yes | No | Yes | Yes | Yes | Ye | No | | Yes | 9 | 81.8% |
| Werthmann et al | Yes | Yes | Yes | Yes | No | Yes | Yes | Yes | Ye | No | | Yes | 9 | 81.8% |
| Tagiyeva et al | Yes | Yes | Yes | Yes | yes | Yes | Yes | Yes | Ye | No | | Yes | 9 | 81.8% |
| Weselak et al | Yes | Yes | Yes | Yes | No | Yes | Yes | Yes | No | Yes | | Yes | 9 | 81.8% |

**JBI checklist for case control studies**

| Studies | Comparability | Case and controls matched properly | Use the same criteria for cases & control | standard, valid and reliable exposure assessment | Exposure measured cases & control similar way | confounding factors identified | strategies for confounding factors stated | Outcomes assessed in a standard, valid and reliable | Enough exposure period | Appropriate statistical analysis used | Total score out of 8 | Level of bias |
| --- | --- | --- | --- | --- | --- | --- | --- | --- | --- | --- | --- | --- |
| Halit et al | Yes | Yes | Yes | Yes | Yes | Yes | No | Yes | Yes | Yes | 9 | 90 % |
| Meng et al | Yes | Yes | Yes | Yes | Yes | Yes | No | Yes | Yes | Yes | 9 | 90% |
| Salam et al | Yes | Yes | Yes | Yes | Yes | Yes | Yes | Yes | Yes | Yes | 10 | 100% |

**JBI for cross sectional studies**

| Studies | Clear eligibility criteria | Description of subject and setting | Valid & reliable method for exposure assessment | Standard criteria used for measurement | Confounding factors identification | Develop strategy for confounding factors | Valid & reliable method to measured outcomes | Appropriate statistical analysis | Total score out of 8 | Level of bias |
| --- | --- | --- | --- | --- | --- | --- | --- | --- | --- | --- |
| Gharibi et al a | Yes | Yes | Yes | Yes | Yes | No | Yes | Yes | 7 | 75% |
| Gharibi et al b | Yes | Yes | Yes | Yes | Yes | No | Yes | Yes | 7 | 87.5% |
| Kuramaus et al | Yes | Yes | Yes | Yes | Yes | Yes | Yes | Yes | 8 | 100% |
| Kuramaus et al | Yes | Yes | Yes | Yes | Yes | No | Yes | Yes | 7 | 87.5% |
| Lu et al | Yes | Yes | Yes | Yes | Yes | Yes | Yes | Yes | 8 | 100% |
| Malaeb et al | Yes | Yes | Yes | Yes | No | No | Yes | Yes | 6 | 75% |
| Masley et al | Yes | Yes | Yes | Yes | No | No | Yes | Yes | 6 | 62.5% |
| Perla et al | Yes | Yes | Yes | No | Yes | No | Yes | Yes | 7 | 87.5% |
| Raherison et al | Yes | Yes | Yes | Yes | Yes | No | Yes | Yes | 7 | 87.5% |
| Runkle et al | Yes | Yes | Yes | Yes | No | Yes | Yes | Yes | 7 | 87.5% |
| Salameh et al | Yes | Yes | Yes | Yes | Yes | Yes | Yes | Yes | 8 | 100% |
| Wang et al 2021 | Yes | Yes | Yes | Yes | Yes | No | Yes | Yes | 7 | 87.5% |
| Xiao et al | Yes | Yes | Yes | Yes | Yes | No | Yes | Yes | 7 | 87.5% |
| Xu et al | Yes | Yes | Yes | No | Yes | No | Yes | Yes | 7 | 87.5% |

Even though synthesis of the evidence from different studies is significantly influenced by the quality evaluation scores for cohort, case-control, and cross-sectional studies, all assessment score of included studies using JBI checklist had higher quality assessment scores indicating a lower risk of bias, more reliable and greater weight in the synthesis of evidence. A higher percentage indicates a higher methodological quality and lower risk of bias present in each study, which in turn assures more reliable evidence synthesis process and drawing conclusion.
